# Comparative Transcriptomics of Fat Bodies between Symbiotic and Quasi-Aposymbiotic Adult Females of *Blattella germanica* with Emphasis on the Metabolic Integration with Its Endosymbiont *Blattabacterium* and Its Immune System

**DOI:** 10.3390/ijms25084228

**Published:** 2024-04-11

**Authors:** Francisco J. Silva, Rebeca Domínguez-Santos, Amparo Latorre, Carlos García-Ferris

**Affiliations:** 1 Institute for Integrative Systems Biology (I2SysBio), University of Valencia and Spanish Research Council, 46980 Paterna, Spain; rebeca.dominguez@uv.es (R.D.-S.); amparo.latorre@uv.es (A.L.); 2 Genomics and Health Area, Foundation for the Promotion of Sanitary and Biomedical Research of the Valencia Region, 46020 Valencia, Spain; 3 Department of Biochemistry and Molecular Biology, University of Valencia, 46100 Burjassot, Spain

**Keywords:** *Blattella germanica*, *Blattabacterium*, fat body, transcriptome, cuticle, tyrosine metabolism, uricolytic pathway, metabolite transporters, peptidoglycan-recognition proteins, antimicrobial peptides

## Abstract

We explored the metabolic integration of *Blattella germanica* and its obligate endosymbiont *Blattabacterium cuenoti* by the transcriptomic analysis of the fat body of quasi-aposymbiotic cockroaches, where the endosymbionts were almost entirely removed with rifampicin. Fat bodies from quasi-aposymbiotic insects displayed large differences in gene expression compared to controls. In quasi-aposymbionts, the metabolism of phenylalanine and tyrosine involved in cuticle sclerotization and pigmentation increased drastically to compensate for the deficiency in the biosynthesis of these amino acids by the endosymbionts. On the other hand, the uricolytic pathway and the biosynthesis of uric acid were severely decreased, probably because the reduced population of endosymbionts was unable to metabolize urea to ammonia. Metabolite transporters that could be involved in the endosymbiosis process were identified. Immune system and antimicrobial peptide (AMP) gene expression was also reduced in quasi-aposymbionts, genes encoding peptidoglycan-recognition proteins, which may provide clues for the maintenance of the symbiotic relationship, as well as three AMP genes whose involvement in the symbiotic relationship will require additional analysis. Finally, a search for AMP-like factors that could be involved in controlling the endosymbiont identified two orphan genes encoding proteins smaller than 200 amino acids underexpressed in quasi-aposymbionts, suggesting a role in the host–endosymbiont relationship.

## 1. Introduction

Long-term associations between animal hosts and microbial symbionts are widespread in nature, and they have been very important in shaping the evolution of many eukaryotic lineages [1,2]. Among insects, most species are involved in some kind of symbiotic association with bacteria [3], which in some cases are mutualistic relationships characterized by fitness advantages [4,5,6]. These symbiotic relationships evolved multiple times for a variety of reasons. One of the most frequent is the ability of an insect species to feed on an unbalanced diet, where some nutrients (some amino acids or vitamins, for example) are present in insufficient amounts [4]. In particular, intracellular endosymbioses are typically nutritional and are based on the establishment of a metabolic integration with the host [7]. In other cases, the microbial symbionts help to detoxify plant compounds [8], enable indigestible food materials to be used as nutrients [9], recycle waste products as uric acid [10], participate in the hardening of the cuticle [11,12], or promote the insect’s health [13].

The German cockroach *Blattella germanica* (Blattodea) is a good model to study these complex symbiotic relationships because two symbiotic systems coexist in a single individual: a bacterial obligate intracellular endosymbiont *Blattabacterium cuenoti* (hereinafter *Blattabacterium*) [14] and a rich and complex gut microbiota [15]. *Blattabacterium*, a Gram-negative bacterium belonging to the phylum Bacteroidetes, is vertically transmitted from mother to offspring through the infection of the oocytes in each generation [16,17,18].

*Blattabacterium* endosymbiosis originated from a single ancestral infection after the divergence of the order Blattodea from Mantodea around 263 million years ago (MYA) [19,20], and they coevolved with their hosts since then [21]. *Blattabacterium* is widely distributed in cockroach lineages, with the exception of the cave-dwelling *Nocticola* [22]. However, among termites (also Blattodea), *Blattabacterium* persists in the lower termites *Mastotermes*, but in the others, it was replaced around 50 MYA by the acquisition of a rich and specialized microbial community and cellulolytic flagellates [19,23,24]. The long-term association of *Blattabacterium* with its host resulted in a severe genome reduction, leading to complete dependence on the intracellular environment [25,26].

*Blattabacterium* is located in the cytoplasm of one type of fat body cell called bacteriocytes [27,28], where it complements the metabolic capacity of the host [29,30]. The other two types of cells present in fat bodies are trophocytes, which are specialized for storing energy compounds, and urocytes, where excess nitrogen is stored as uric acid to provide a nitrogen resource for amino acid synthesis [28].

The fat body is involved in the metabolism of basic energy compounds, accumulating reserves and mobilizing them during metamorphosis or various stress situations such as starvation [31], and is responsible for innate and acquired immunity by producing effector molecules such as antimicrobial peptides (AMPs), which are released to the hemolymph [32,33]. Moreover, the fat body has an important role in the metabolism of amino acids, contributing, for example, to regulating the tyrosine content in the hemolymph, which participates in cuticle sclerotization [34] and is responsible for the synthesis of the majority of storage proteins that circulate in the hemolymph, as hexamerins, which act as tyrosine and phenylalanine reservoirs and transporters and can be incorporated into nascent cuticle [35,36].

The analysis of the gene repertoires of seven *Blattabacterium* genomes (reviewed in [15]) revealed that, in general, the endosymbionts display complete or almost complete pathways for the ten-insect essential amino acids (including phenylalanine) and six non-essential ones (including tyrosine) [26]. However, the most striking result was the retention of the complete urea cycle, plus the genes encoding urease (*ureAB* and *ureC*) [10], which degrades urea and produces ammonia as an end product. This pathway is complemented by the host mobilizing the uric acid from the neighboring urocytes to produce urea through a five-step pathway [26]. Urea is then degraded into ammonia and CO_2_ by the endosymbiotic urease. Ammonia can be incorporated into glutamate and glutamine by the endosymbiont and the host, respectively, participating in the production of essential amino acids for the host [29,30].

Each *Blattabacterium* cell, with its prokaryotic membranes, is located in the bacteriocyte inside a vacuole of eukaryotic origin, called symbiosome, surrounded by the symbiosomal membrane, which acts as an interface between the endosymbiont and the bacteriocyte cytoplasm [27]. To enable metabolic integration, this eukaryotic/prokaryotic interface must actively control the traffic of metabolites in both directions, allowing nutrients to be supplied to the endosymbiont and specific biosynthetic products to be exported to the host, as it has been proposed in other symbioses in insects [37]. The in silico reconstruction of *Blattabacterium* metabolism from *B. germanica* led to the conclusion that several metabolites must be transported across the eukaryotic/prokaryotic interface to allow metabolic integration [38,39]. To achieve this objective, specific transporters of eukaryotic and prokaryotic origin are required, which should be located, respectively, in the eukaryotic and prokaryotic membranes of the interface [37].

Currently, no eukaryotic transporters that allow the trafficking of specific metabolites across the symbiosomal membrane in the *B. germanica*-*Blattabacterium* system has been identified. On the other hand, *Blattabacterium* genome erosion led to the loss of many genes [40,41], including most substrate-specific prokaryotic transporters, making the capabilities to transport metabolites through the endosymbiont membranes very limited [10], which constitutes a common characteristic of endosymbiosis in insects [30,42,43]. In this context, it has been proposed that symbiotic AMP-like factors that are produced by eukaryotic hosts during the symbiotic interaction and are consequently lineage-specific could be targeted to the endosymbiont membranes, transitorily increasing their permeability and allowing the traffic of metabolites through these membranes without the need for specific transporters encoded by the endosymbiont genome [44,45]. Some of these AMP-like factors could even enter the bacteria and affect the physiology of the endosymbiont by controlling the bacterial load and preventing it from escaping from the bacteriocyte and becoming an infection in other tissues [46,47,48].

Recently, we focused on characterizing the AMP gene repertoire harbored in the *B. germanica* genome. We found 39 AMP genes, corresponding to the expansion of five gene families [49]. These AMP genes were expressed in many tissues, but the highest expression levels were detected in salivary glands and in hemolymph [50]. In fact, although only some AMP genes were described as being expressed in the fat body, they should not affect the population of the bacterial endosymbiont located in the bacteriocytes. In addition, some AMPs may be considered symbiotic AMPs, performing functions related to the control of the endosymbiont population. For example, the relationship between coleoptericin A and the control of a bacterial endosymbiont has been reported in weevils of the genus *Sitophilus*, where it targets the endosymbionts, inhibiting bacterial cell division and leading to giant bacterial cells [46]. Another way, described in several insects, is related to the expression in bacteriocytes of a peptidoglycan recognition protein (PGRP), PGRP-LB, which negatively modulates the immune deficiency (IMD) pathway by cleaving bacterial peptidoglycan into non-immunogenic fragments [51,52]. Finally, in the *Acyrthosiphon pisum*-*Buchnera aphidicola* endosymbiosis, a new class of proteins, bacteriocyte-specific cysteine-rich peptides, was identified that exhibit antimicrobial activity in vitro against *Escherichia coli*. These AMP-like factors are lineage-specific and could be involved in endosymbiosis, including bacteriocyte homeostasis and endosymbiont control [53,54,55].

Antibiotic treatment is a powerful tool to selectively eliminate bacteria. In our laboratory, we treated *B. germanica* with various antibiotics [56]. Rifampicin was the only one that affected *Blattabacterium*, and it was used to generate aposymbiotic individuals [57,58,59]. However, the reduction of the endosymbiont population is observable not in the treated generation but in its progeny, where it is reduced by up to five orders of magnitude [57], producing quasi-aposymbiotic individuals [58,59]. This is because *Blattabacterium* is susceptible to rifampicin in adults only during its extracellular phase in the ovaries when it leaves the protection of the bacteriocytes to infect the mature oocytes [16]. The role of the endosymbiotic population is so essential for the host that the gut microbiota in *B. germanica* could not compensate for it, and the fitness of quasi-aposymbiotic individuals was drastically affected [58,59]. The metabolic analysis carried out in the pioneering studies with aposymbiotic *B. germanica* made it possible to infer the contribution of the endosymbiont to fat body function. Aposymbiotic individuals were unable to synthesize several amino acids, including tyrosine, an essential precursor in the tanning process, which could explain their lighter coloration. *Blattabacterium* was also related in these studies to the recycling of stored urates because the elimination of endosymbiont resulted in the accumulation of uric acid crystals in the fat body [60]. Finally, the comparison of symbiont-bearing and aposymbiotic populations can be used to assess the influence of the symbiont population on the metabolic and physiological processes of the host and the mechanisms by which the metabolic integration of host and endosymbiont is established and maintained [51,61,62,63].

In this study, we compared fat body gene expression in quasi-aposymbiotic and symbiotic (control) adults of *B. germanica* with the aim of searching for transcripts differentially expressed in the absence of *Blattabacterium* that may be relevant to understanding the endosymbiont–host interaction. We also wanted to know whether the extreme reduction of the endosymbiont population may drive the host to regulate those metabolic pathways that are complemented by the endosymbiont metabolism in control individuals.

## 2. Results

### 2.1. Differential Gene Expression between Quasi-Aposymbiont and Control Fat Bodies

We isolated RNA from fat bodies of adult females, four replicates (each a pool of three) from a quasi-aposymbiotic population, and another four from a control population. The quasi-aposymbionts were obtained after the treatment of the adults of the previous generation with the antibiotic rifampicin (see Introduction and Materials and Methods). RNA was used to synthesize paired-end Illumina libraries. RNA-seq reads were filtered and used for a differential expression analysis. To help in the analysis, we first obtained a de novo transcriptome assembly with these 8 samples and 24 additional samples from other tissues (see Material and Methods). This transcriptome was changed, removing those transcripts of very low expression and replacing AMP genes and IMD pathway genes with those characterized in *B. germanica* in previous studies [49,50].

Transcripts with differential gene expression were obtained with DESeq2. It produced 359 transcripts that were overexpressed and 460 that were underexpressed in quasi-aposymbionts compared to controls (Table 1).

We searched for transcripts derived from *Blattabacterium* genes and detected 13 transcripts that were underexpressed in quasi-aposymbionts but none that were overexpressed. Although RNA samples were enriched in eukaryotic mRNA with poly(A)-tail capture for Illumina sequencing, this process was not completely effective, and some transcripts corresponding to highly expressed genes (rRNA genes or the *GroES*-*GroEL* operon) were still detected.

The TransDecoder program with a minimum length of 60 codons was used to identify encoded proteins in these transcripts. Most transcripts were potentially coding, 246 of those were overexpressed and 342 of those were underexpressed (excluding those from *Blattabacterium*), whereas 113 and 105 were annotated as non-coding (Table 1). Predicted proteins (in some transcripts more than one hypothetical protein was predicted) were annotated using eggNOG-mapper with default conditions. The program reported several functional annotations, orthology assignments, and domain predictions, such as COG categories, KEGG_ko, GO terms, PFAM domains, etc. Only the proteins of 160 overexpressed and 227 underexpressed transcripts were assigned annotations (Table 1). Those without functional assignments were used as query proteins in a BLASTP against non-redundant protein sequences (nr) database from NCBI (E-value cutoff 10^−5^). It added 27 and 55 transcripts to those with eggNOG annotations (Table 1). Transcripts and encoded protein annotations are found in Table S1.

**Figure 1:**
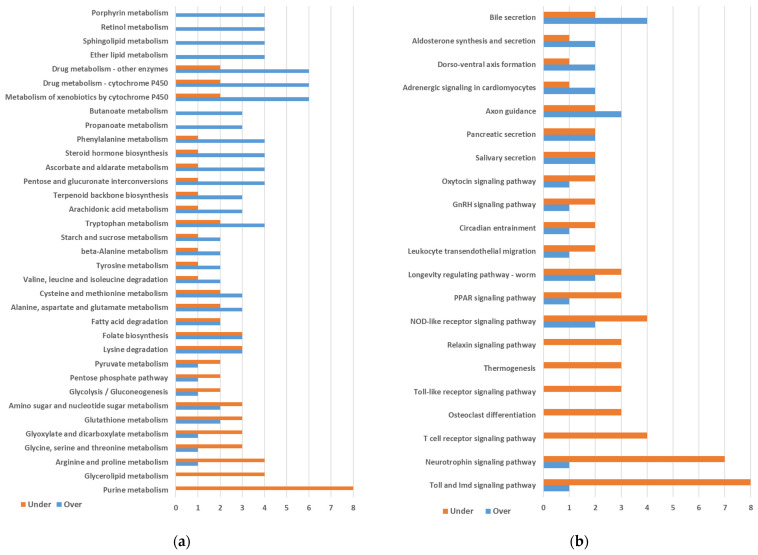
KEGG maps involved in (**a**) Metabolism and (**b**) Organismal Systems. Only maps containing 3 or more differentially expressed genes are included. Maps are ordered based on the difference between the numbers of overexpressed minus underexpressed genes (from top to bottom). Transcripts from the same gene were grouped for plotting.

**Figure 2:**
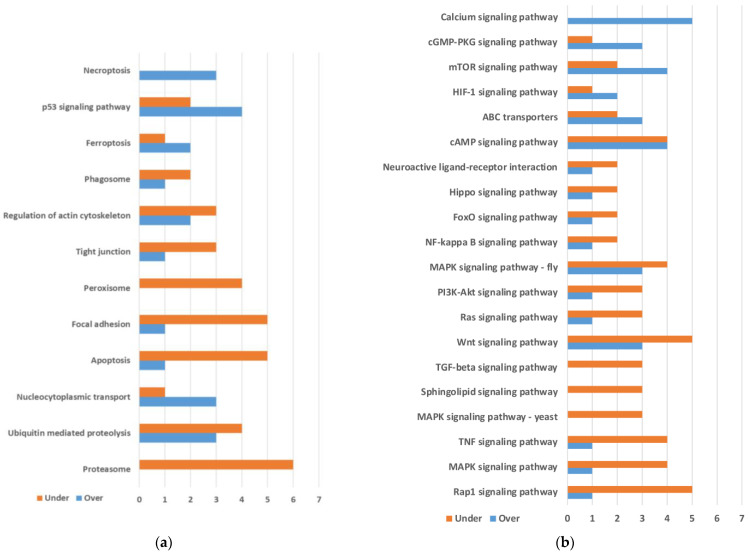
KEGG maps involved in (**a**) Genetic Information Processing and Cellular Processes. (**b**) Environmental Information Processing. Only maps containing 3 or more differentially expressed genes are included. Maps are ordered based on the difference between the numbers of overexpressed minus underexpressed genes (from top to bottom). Genetic Information Processing (the bottom three maps) and Cellular Processes were ordered separately. Transcripts from the same gene were grouped for plotting.

KEGG level C maps with three or more differentially expressed genes were plotted for Metabolism (Figure 1a), Organismal Systems (Figure 1b), Genetic Information Processing (Figure 2a), Cellular Processes (Figure 2a), and Environmental Information Processing (Figure 2b).

In the category of Metabolism, we found several maps containing more over- than underexpressed genes. The maps with the highest numbers of overexpressed genes were three related to the metabolism of xenobiotics and drugs. The maps corresponding to Porphyrin metabolism, Retinol metabolism, Sphingolipid metabolism, and Ether lipid metabolism also showed a high number of overexpressed genes. However, all these seven maps were identified due to the presence of the same four genes corresponding to a single enzymatic activity. We also observed four overexpressed genes in the Phenylalanine metabolism, an important pathway that may be involved in cuticle synthesis. On the other hand, we observed mainly underexpressed genes in Arginine and proline metabolism, Glycerolipid metabolism, and especially Purine metabolism (Figure 1a).

Only a few maps showed more over- than underexpressed genes in the category of Organismal Systems (Figure 1b). On the contrary, most maps preferentially displayed underexpressed genes, such as the Neurotrophin signaling pathway map included in Nervous System (KEGG level B) and the Toll and Imd signaling pathway map included in Immune System (KEGG level B). For the latter map, eggNOG assigned ko annotations to five underexpressed genes (two PGRPs; JUN, transcription factor AP-1; IRAK1, interleukin-1 receptor-associated kinase 1; NFKBIA, NF-kappa-B inhibitor alpha) and one overexpressed (DUOX, dual oxidase). However, we detected, after BLASTP analyses, that eggNOG missed three additional underexpressed AMP genes for this map (*defensin_g9*, *defensin_g10*, and *termicin_g4*). The probable reason is that they are genes of small sizes. They are included in Figure 1b and will be discussed later.

In Genetic Information Processing, only three maps displayed three or more differentially expressed transcripts. Proteasome was the most relevant, with six underexpressed genes (Figure 2a). In Cellular Processes, some pathways involved in cell growth and death, such as Necroptosis, p53 signaling pathway, or Ferroptosis, displayed more over- than underexpressed genes, while others, such as Apoptosis, Focal adhesion, or Peroxisome, involved mainly underexpressed genes (in Figure 2a, maps for Genetic Information Processing and maps for Cellular Processes are represented together). In Environmental Information Processing, a preponderance of signaling pathways with more underexpressed than overexpressed genes was observed (Figure 2b).

To explore some of these maps with differentially expressed genes, several functions were analyzed in greater depth in order to understand the importance of both the presence and the absence of the endosymbionts for the insect’s metabolism and physiology.

### 2.2. Synthesis of Tyrosine and Catechol Derivatives for Cuticle Sclerotization

Among the transcripts significantly overexpressed in quasi-aposymbiont versus control fat bodies, several were involved, or potentially involved, in cuticle sclerotization and especially in the synthesis of tyrosine and catecholamine precursors for the cross-linking of adult cuticle proteins (Table 2).

**Figure 3:**
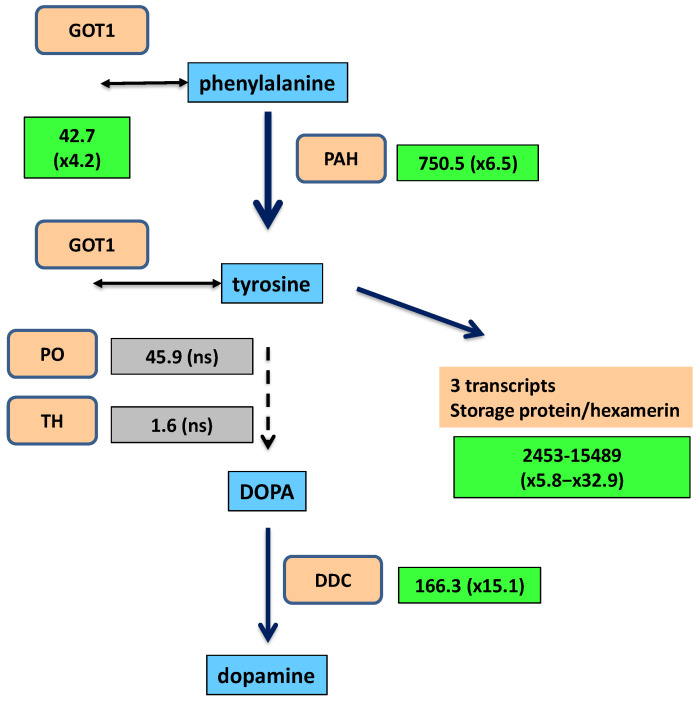
Overexpression of genes involved in cuticle sclerotization and pigmentation in quasi-aposymbiotic fat bodies. Green rectangles include the average TPM value of four samples and, in parentheses, the fold change of the ratio of quasi-aposymbiont/control fat bodies. Values in green rectangles are significant (*p*-value < 0.05) and have a fold change higher than 2. For two or more transcripts, the ranges of TPM value and fold change are shown. Grey rectangles show values that do not fulfill the previous two criteria (marked as “ns”). Blue and orange rectangles are metabolites and proteins, respectively. GOT1 (aspartate aminotransferase, EC 2.6.1.1); PAH (phenylalanine-4-hydroxylase, EC 1.14.16.1); PO (phenoloxidase_subunit_1); TH (tyrosine 3-monooxygenase, EC 1.14.16.2); DDC (dopa decarboxylase, EC 4.1.1.28).

A gene collateral to the pathway, encoding an aspartate aminotransferase (GOT1; EC 2.6.1.1), was overexpressed (fold change 4.2). It is potentially involved in the transfer of the amino group to synthesize phenylalanine and tyrosine. The pathway for the synthesis of dopamine from phenylalanine was strongly upregulated in quasi-aposymbionts (Figure 3). The first enzyme, phenylalanine 4-monooxygenase or phenylalanine hydroxylase (PAH), is encoded by one of the ten highly expressed genes with differential overexpression in quasi-aposymbionts. The gene encoding the third enzyme, aromatic amino acid decarboxylase or dopa decarboxylase (DDC), is not only one of the highly expressed genes in quasi-aposymbionts but is 15.1-fold overexpressed compared with control fat bodies. To avoid confusion with other paralogous genes, such as tyrosine decarboxylase, histidine decarboxylase, or 3,4-dihydroxylphenylacetaldehyde synthase [64], a phylogenetic analysis was performed, confirming its correct annotation as DDC (Figure S1).

To explain the enzymatic gap in the pathway to dopamine in fat bodies (from tyrosine to L-DOPA), a screening of genes encoding enzymes potentially involved in this step was performed. The expression of tyrosine 3-monooxygenase (ENA: PSN41889.1) (TH) was detected, but it was almost null (1.6 TPM, transcripts per million) and not differentially expressed. Another gene, phenoloxidase_subunit_1 (ENA: PSN38098.1), showed moderate expression (45.9 TPM) but was also not differentially expressed (Figure 3). These results lead us to consider the possibility that *B. germanica* PAH is a multifunctional enzyme able to hydroxylase both phenylalanine and tyrosine, although other reasons may explain why an aromatic amino acid decarboxylase is highly and differentially expressed in quasi-aposymbiotic fat bodies.

**Figure 4:**
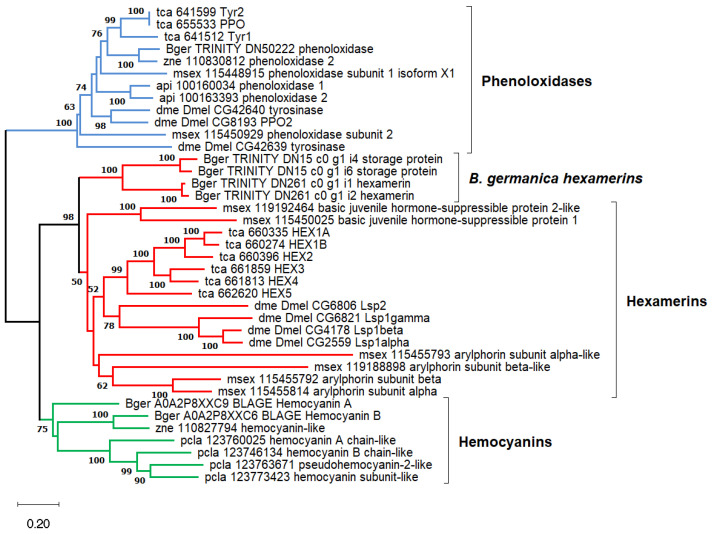
Phylogenetic reconstruction of phenoloxidases (blue), hemocyanins (green), and hexamerins (red) in several arthropods. Proteins were selected from KEGG genes, except those of *B. germanica* (Bger). Abbreviated taxonomic codes were api (*Acyrthosiphon pisum*, pea aphid), dme (*Drosophila melanogaster*, fruit fly), msex (*Manduca sexta*, tobacco hornworm), pcla (*Procambarus clarkia*, red swamp crayfish), tca (*Tribolium castaneum*, red flour beetle), and zne (*Zootermopsis nevadensis*, dampwood termite). The phylogenetic tree was inferred with the Neighbor-Joining method, the JTT evolutionary model, and 1000 bootstrap replicates on an amino acid alignment of 456 residues. Bootstrap values smaller than 50 are not shown. The scale bar corresponds to 0.20 estimated amino acid substitutions per site.

The production of tyrosine will be partially directed to the synthesis of hexamerins, a type of protein present in high concentrations in the hemolymph of insects [35]. These proteins provide amino acids to build adult structures or may be directly incorporated into the cuticle [36]. Three transcripts grouped by the program TRINITY as isoforms of the gene DN15_c0_g1 (“TRINITY_” was removed at the start of the transcript names through the text) displayed the highest level of expression among the differentially overexpressed genes of quasi-aposymbionts (TPM values > 2000). They were overexpressed in a range of 5.8 to 32.9. The comparison of the three transcripts suggests the presence of at least two different genes represented by DN15_c0_g1_i4 (ENA: PSN36665.1) and DN15_c0_g1_i6 (no ENA orthologous gene) that display a 10% difference at the nucleotide level in the coding sequence. However, several signs, such as the high expression or the detection of other related transcripts not differentially expressed, suggest a potentially larger gene family. These proteins may be considered hexamerins, storage proteins that belong to an ancestral gene family composed of phenoloxidases, hemocyanins, and hexamerins. A phylogeny inferred from several insect proteins and a few crustacean hemocyanins was obtained to confirm its position in the gene family (Figure 4). Among *B. germanica* fat body transcripts, two types of hexamerin transcripts were detected. Both encode proteins with a high frequency of the amino acid tyrosine: DN15_c0_g1 (10%) (already mentioned) and DN261_c0_g1 (15%). Only DN15_c0_g1 transcripts were differentially overexpressed in quasi-aposymbionts.

### 2.3. Uric Acid and Purine Metabolism

**Figure 5:**
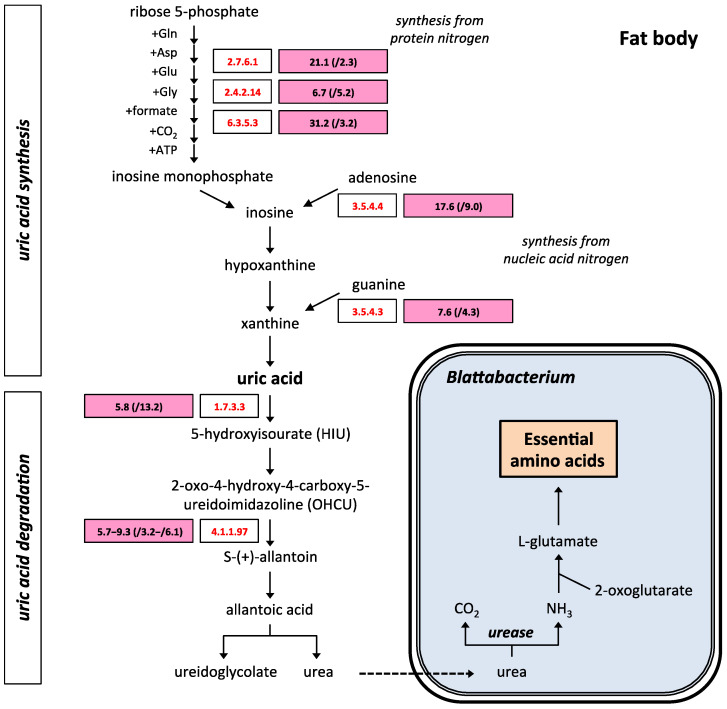
Metabolism of uric acid in quasi-aposymbiotic fat bodies. Biosynthesis of uric acid is produced de novo using amino acids from protein degradation or using purines obtained from nucleic acids turnover. Uric acid is degraded to urea via the uricolytic pathway. Pink rectangles contain the average TPM values of four samples from quasi-aposymbiotic fat bodies and, in parentheses, the underexpression fold change compared to the control. Values in pink rectangles are significant (*p*-value < 0.05) and have a fold change higher than 2. For two or more transcripts, the ranges of TPM value and fold change are shown. For each step with differential gene expression, the EC number is shown: ribose-phosphate pyrophosphokinase (EC 2.7.6.1), amidophosphoribosyltransferase (EC 2.4.2.14), phosphoribosylformylglycinamidine synthase (EC 6.3.5.3), adenosine deaminase (EC 3.5.4.4), guanine deaminase (EC 3.5.4.3), urate oxidase (EC 1.7.3.3), and 2-oxo-4-hydroxy-4-carboxy-5-ureidoimidazoline decarboxylase (EC 4.1.1.97).

Purine metabolism was the metabolic pathway with the highest number of genes underexpressed in quasi-aposymbiotic fat bodies (Figure 1a). The most relevant finding was the underexpression of seven genes involved in the synthesis of uric acid and its degradation to urea. Ribose-phosphate pyrophosphokinase (EC 2.7.6.1), amidophosphoribosyltransferase (EC 2.4.2.14), and phosphoribosylformylglycinamidine synthase (EC 6.3.5.3) are encoded by three genes underexpressed in the biosynthetic pathway, which synthesize uric acid de novo using amino acids from protein degradation, and this is the most important pathway responsible for the synthesis of urates in insects [65]. Adenosine deaminase (EC 3.5.4.4) and guanine deaminase (EC 3.5.4.3) are encoded by two underexpressed genes involved in the synthesis of uric acid using purines obtained from nucleic acid turnover. Finally, in the uricolytic pathway, which degrades uric acid to urea, the genes for urate oxidase (EC 1.7.3.3) and 2-oxo-4-hydroxy-4-carboxy-5-ureidoimidazoline decarboxylase (EC 4.1.1.97) are underexpressed (Figure 5). The genes encoding the other enzymes of the uricolytic pathway were also expressed, but their expression was not significantly different (*p*-value < 0.05) and/or had a fold change smaller than 2 compared to control fat bodies. The low titer of *Blattabacterium* cells explains the downregulation of the pathway to produce urea, which cannot be degraded to ammonia.

### 2.4. Metabolite Transporters

Metabolite transport in the bacteriocyte is essential to establish and maintain metabolic integration between host and endosymbiont. For this reason, a controlled influx and efflux of metabolites through the cell membrane and the symbiosomal membrane of the bacteriocyte must be established. To identify transporters that could be responsible for this traffic of metabolites in the *Blattabacterium*-*B. germanica* endosymbiosis, we screened the differentially expressed transcripts in search of genes underexpressed more than five-fold in fat bodies of quasi-aposymbionts and encoding integral membrane proteins with more than three TMSs (transmembrane segments) according to DeepTMHMM analysis. These proteins are possible candidates for participation in the transport of metabolites through the cell membrane of the bacteriocyte or the symbiosomal membrane. Thirteen proteins were identified as candidates for membrane transporters involved in endosymbiosis (Table 3). The cellular localization of these candidate proteins was analyzed using DeepLoc, and the possible function of the transporters was deduced from the best hits found in the BLASTP analysis against the Transporter Classification Database (TCDB).

Seven of the identified proteins are localized to the cell membrane, six of them being proteins similar to transporters of the Major Facilitator Superfamily (TC# 2.A.1), with the following transporter families. Two proteins (encoded by DN12960_c0_g1_i2 and DN4893_c0_g1_i1) show similarity to glutamate or phosphate transporters that are members of the Anion:Cation Symporter Family (TC# 2.A.1.14), while two other proteins (encoded by DN1327_c1_g1_i8 and DN2708_c1_g1_i2) are similar to trehalose transporters that are members of the Sugar Porter Family (TC# 2.A.1.1). These results indicate that the transport of trehalose and glutamate or phosphate (with a 20.9-fold change for DN12960_c0_g1_i2) must be very important for the maintenance of the endosymbiosis. Another protein (encoded by DN22022_c0_g2_i1) is similar to some members of the Unidentified Major Facilitator-14 Family (TC# 2.A.1.65), whose function is unknown.

The transcript DN9218_c0_g1_i4 encodes a protein that has a cell membrane|endoplasmic reticulum localization and presents similarity to the ATP binding domain of ABC membrane transporters that are members of the Eye Pigment Precursor Transporter Family (TC# 3.A.1.204).

Two transcripts encode membrane proteins that localize to cell membrane|lysosome/vacuole. The protein encoded by DN1382_c0_g1_i1 shows similarity to members of the Zinc (Zn^2+^)-Iron (Fe^2+^) Permease Family (TC# 2.A.5). On the other hand, the protein encoded by DN2082_c0_g1_i1 shows similarity to members of the Urea Transporter Family (TC# 1.A.28). This urea transporter could participate in the transport of urea, a key metabolite in metabolic integration in the symbiosis, through the cell membrane participating in the movement of urea between urocytes and bacteriocytes, or in the symbiotic vacuole importing urea for its degradation by the endosymbiont.

Finally, two transcripts (DN74365_c0_g1_i1 and DN8_c1_g1_i10), which encode membrane proteins with four TMSs and a lysosome/vacuole predicted localization, are underexpressed in quasi-aposymbionts (especially DN8_c1_g1_i10 with a 22.3-fold change). The analysis by BLASTP against the TCDB indicated that these proteins showed similarity to members of the 4 TMS Multidrug Endosomal Transporter Family (TC# 2.A.74), with unknown functions.

### 2.5. Immune System and Antimicrobial Peptides

The eggNOG analysis revealed the underexpression of several genes in the Toll-Imd pathways in quasi-aposymbionts (Table 4). This was the case for the *cactus*, *pelle*, PGRP, and AMP genes. One of the transcripts of the *cactus* gene was only expressed in control fat bodies, while the two other transcripts displayed a fold change of around 5 compared to control fat bodies. The other gene, *pelle*, showed a slight underexpression in quasi-aposymbionts (fold change of 2.3).

Two transcripts were annotated by eggNOG as encoding PGRPs were underexpressed in quasi-aposymbionts. DN3036_c0_g1_i2 and DN3159_c1_g1_i1 encode small proteins of 182 and 226 amino acids, respectively (Table 4). Using the PGRP domain superfamily (IPR036505) obtained from these two transcripts as a query in a TBLASTN search against our transcriptome assembly, we were able to identify 12 PGRP-like genes. Some of these genes showed alternative isoforms. The DN3036_c0_g1 gene showed an alternative isoform 1, encoding a protein of 237 amino acids with very low expression (Table 4), which differed from isoform 2 in the N-terminal end. SignalP only predicted a signal peptide in isoform 1 (residues 1–19), thus suggesting the extracellular localization of the former and the cytoplasmatic localization of the latter, although the DeepLoc program predicted both cytoplasm|extracellular locations for isoform 2. The protein encoded by DN3159_c1_g1_i1 was located at the cell membrane by this latter program.

In order to classify these two PGRP proteins, we performed a phylogenetic analysis comparing them with those reported in other insect species. We decided to include in the phylogeny the 191-amino acid protein encoded by the non-differentially expressed transcript DN4891_c0_g1_i1 because it was quite similar to DN3036_c0_g1_i2 (54%). The protein encoded by DN4891_c0_g1_i1 displays a signal peptide (residues 1−21), and the DeepLoc-predicted localization was extracellular (Table 4).

**Figure 6:**
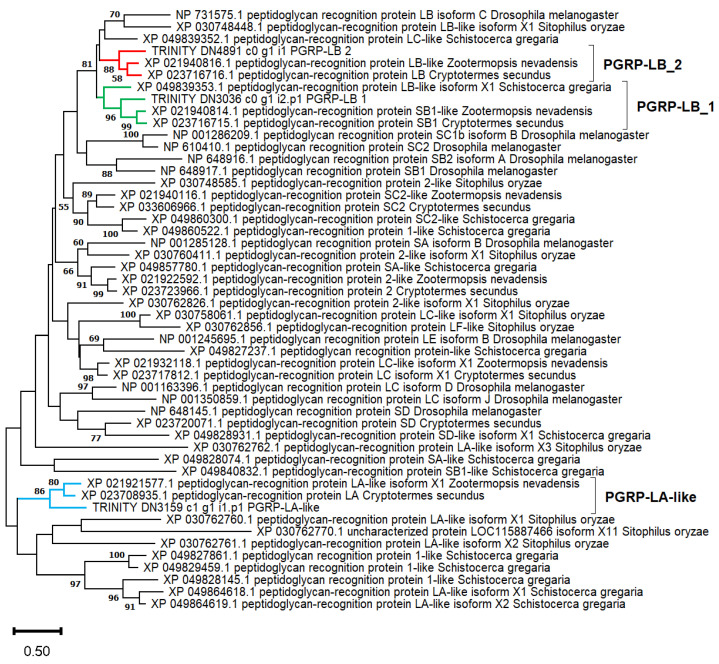
Phylogenetic analysis of PGRP proteins. Maximum likelihood phylogeny: evolutionary model LG + G, using all sites and 1000 bootstrap replicates. The clades for PGRP-LB_2 (red), PGRP-LB_1 (green) and PGRP-LA-like (blue) are shown. Bootstrap values higher than 50 are shown near the corresponding nodes. The root of the tree was placed at the mid-point. The scale bar corresponds to 0.50 estimated amino acid substitutions per site.

The selected *B. germanica* PGRP proteins were aligned with reference proteins of *Zootermopsis nevadensis* and *Cryptotermes secundus* (Blattodea), *Schistocerca gregaria* (Orthoptera), *Sitophilus oryzae* (Coleoptera), and *D. melanogaster* (Diptera). A maximum likelihood phylogenetic analysis based on a short alignment of 178 amino acid sites, including most of the IPR036505 domain superfamily, was obtained (Figure 6). Although most nodes were not supported by significant bootstrap values, the tree supports the existence of two types of PGRP-LB genes in Blattodea and Orthoptera (both Polyneoptera) and one in Coleoptera and Diptera. We annotated the genes DN3036_c0_g1 and DN4891_c0_g1 as *PGRP-LB_1* and *PGRP-LB_2*, respectively. BLASTP searches against the proteome of *B. germanica* [66] showed that DN3036_c0_g1_i1 (the isoform non-differentially expressed) was identical to PSN41495.1 (Peptidoglycan-recognition protein LB, gene = *PGRP-LB_0*), while DN4891_c0_g1 was identical to PSN41491.1 and PSN41492.1 (Peptidoglycan-recognition protein LB, gene = *PGRP-LB_2*).

An analysis of the five isoforms of the LB-like protein from *S. oryzae* showed that a signal peptide is present in only three of them (isoforms X3, X4, and X5). All the PGRP-LB proteins from the analyzed termites displayed signal peptides.

Finally, DN3159_c1_g1_i1 was annotated as *PGRP-LA-like* because it was in a clade with the two termite proteins annotated as LA or LA-like. The phylogeny also associated these proteins with those of other insects mainly annotated as PGRP-LA-like but with non-significant bootstrap values (see the bottom of the tree).

Because the control of some insect endosymbionts in the bacteriocytes has been related to the expression of some AMP/AMP-like genes, it would be expected that they were underexpressed in quasi-aposymbionts. In fact, we identified two AMP genes, *defensin_g9* and *defensin_g10* [49] (Table 4), which displayed fold changes of 7 and 8.5, respectively. Both proteins contain signal peptides in the N-terminus and the Defensin_2 domain (PF01097) in the C-terminus. Neither of them was annotated by eggNOG, probably due to their small lengths and low amino acid identities. Although both are expressed in fat bodies, their main site of expression is hemolymph, with a level of expression or around two orders of magnitude higher than in fat bodies [50]. In addition, a new termicin gene (*termicin_g4*), not previously described [49,50], was detected among those transcripts not annotated by eggNOG (Table 4). BLASTP searches identified similar proteins in several species of termites. The protein encoded by *termicin_g4* is 61 amino acids long and contains a signal peptide in the N-terminus and the Toxin_37 domain (PF11415) in the C-terminus. Its amino acid sequence is relatively divergent from those of the other three *B. germanica* termicin genes (47–49% identical). The nucleotide sequence of the gene was annotated in the *B. germanica* genome as C0J52_19948, but the open reading frame annotated in the genome was incorrect [66]. Using RNA seq data from hemolymph and six tissues of adult female *B. germanica* [50], we determined that the main site of expression of this gene was hemolymph, and its expression was around 2–3 orders of magnitude higher than in the other six tissues analyzed, including the fat body (Figure S2).

To detect AMP-like (non-canonical AMP) lineage-specific genes among the underexpressed genes, we extracted transcripts potentially encoding proteins with a maximal length of 200 amino acids that were underexpressed more than five-fold. This produced a set of 70 proteins. Considering that most AMPs contain cysteines, we selected those proteins that contained at least six cysteines. This reduced the set to 14 proteins (Table 5), which included the 3 previously mentioned AMP genes (*defensin_g9*, *defensin_g10*, and *termicin_g4*). Then, these 14 proteins were filtered using several criteria.

The existence of a signal peptide in the AMP-like factor was considered necessary for these proteins to be directed outside the cell or targeted to the lumen of the vacuole/symbiosome. An InterProScan analysis was performed to identify potential signal peptides and protein domains, while DeepLoc analysis was used to predict cellular localizations. Eight of the listed proteins present a signal peptide and an extracellular localization prediction. Three of them correspond to the proteins defensin_g9, defensin_g10, and termicin_g4 (encoded by DN4880_c0_g1_i1), which were ruled out as AMP-like factor candidates for involvement in endosymbiosis, as they are canonical AMPs and have low expression levels compared to hemolymph ([50] and Figure S2 for *termicin_g4*).

In addition to defensin_g9, defensin_g10, and termicin_4, five other proteins with signal peptides and extracellular predictions were found. Their potential functions were identified after BLASTP analysis; DN12569_c0_g1_i1 encodes a protein similar to an adhesive plaque matrix protein from *Z. nevadensis* and, therefore, with a function not directly connected to endosymbiosis; DN2464_c0_g1_i3 encodes a protein that showed similarity to lysozyme c-1 (PSN47213.1) from *B. germanica*, lysozymes being well-known immune effectors in insect innate immunity with antibacterial properties [33]; DN3965_c0_g1_i1 encodes a protein with homology to a hypothetical protein found only in the Blattodea lineage; and finally, DN48391_c0_g1_i1 and DN17653_c0_g1_i2 encode two proteins similar to a hemolymph polysaccharide-binding protein, C-type lectin. Due to the lectin-associated antimicrobial activity and its link between the immune system and the homeostasis of the gut microbiota [67], these two candidate proteins demonstrate special potential.

One of the identified proteins, encoded by DN7034_c0_g1_i1, contains a signal peptide, but the protein is localized to the cell membrane according to DeepLoc analysis. BLASTP analysis indicates that the protein shows similarity to hypothetical proteins of the Sleepless type, which are very widespread in insect lineages. These proteins are glycosylphosphatidylinositol-anchored to the membrane and participate in the regulation of the voltage-gated potassium channel that regulates sleep. Moreover, BLASTP analysis of this protein against the TCBD also indicates that it has homology with members of the Quiver/Sleepless/Dreammist Family (TC# 8.A.231). For this reason, it was discarded as an AMP-like factor candidate.

The five remaining proteins on the list do not contain signal peptides. One of them, encoded by DN5401_c0_g2_i1, has an extracellular localization based on DeepLoc predictive analysis; even DeepTMHMM analysis predicts one TMS in the N-terminal region. Moreover, no homology was identified in the BLASTP analysis. This small orphan protein (only 67 amino acids) should be included in the list of candidates for AMP-like factors involved in endosymbiosis on the basis of its extracellular localization and in the high proportion of cysteine residues (7 out of 67) and the underexpression level observed in the fat body of quasi-aposymbionts (fold change of 47.1); it is the transcript with the greatest change in expression out of all 14 candidates. The rest of the proteins without signal peptides are ruled out as AMP-like factor candidates as follows. Two proteins are predicted to be intracellular by DeepLoc analysis, localized either in mitochondria (protein encoded by DN43375_c0_g1_i1) or in cytoplasm/nucleus (protein encoded by DN54153_c0_g1_i1). The other two proteins (encoded by DN74365_c0_g1_i1 and DN8_c1_g1_i10) presented membrane localization with four TMSs according to DeepTMHMM analysis, and lysosome/vacuole localization according to DeepLoc, corresponding to previously identified transcripts that code for membrane proteins with similarity to transporters of the 4 TMS Multidrug Endosomal Transporter Family (TC# 2.A.74).

For the reasons set out above, the list of candidates for AMP-like factors involved in the homeostasis of endosymbiosis is reduced to five proteins encoded by DN17653_c0_g1_i2, DN2464_c0_g1_i3, DN3965_c0_g1_i1, DN48391_c0_g1_i1, and DN5401_c0_g2_i1. Additionally, it bears mentioning that the proteins encoded by DN3965_c0_g1_i1 and DN5401_c0_g2_i1 seem to be orphans, with no domain identified in the InterProScan analysis or clear result in the BLASTP analysis.

## 3. Discussion

The mutualistic relationship between the order Blattodea and *Blattabacterium* started after its divergence from Mantodea around 263 MYA [16,17]. This association allowed the adaptation of these insects to different environments and to cover potential dietary deficits, either for short periods or for the complete life cycle. To stabilize this association, specific cells, called bacteriocytes, evolved in the fat body. In *B. germanica*, single bacteriocytes are scattered in visceral fat bodies in the abdomen [68]. Their dispersed localizations avoid their isolation from the remaining fat body cells. However, treatment with antibiotics allows a comparative analysis between quasi-aposymbiotic and control fat bodies.

In this study, we used RNA-seq differential expression analysis, to determine and compare the transcriptomic profiles of quasi-aposymbiont and control fat bodies in order to identify genes and pathways involved in the establishment and maintenance of the endosymbiosis and in the response of the host to the absence of endosymbiont. A potential limitation of the study is that we have not used RT-qPCR to validate the results, but recent research supports the view that RNA-seq results are robust and do not always require independent verification [69,70]. Accordingly, the validation of RNA-seq data with RT-qPCR has been declining over the past few years [71]. RT-qPCR-based validation has historically been necessary with microarray analysis; however, RNA-seq technology does not present the same problems as microarrays, and the added value of this validation practice was questioned recently [69,70,71]. RNA-seq is very reproducible and highly accurate for quantifying expression levels [72,73]. Moreover, recent studies indicate that there is a high correlation between RNA-seq and RT-qPCR data. Around 15–20% of the genes showed non-concordant gene expression results between RNA-seq and RT-qPCR data, but only 1.8% of genes were non-concordant with a fold change > 2, these being mainly shorter and lower expressed genes [69,70]. These differences could be due to several factors, such as the level of expression, sensitivities of the assays, and differences in efficiency between PCR primers [70,74]. The filters applied in our study reduced the possibility that we were working with non-concordant genes. We selected transcripts with fold change > 2 and a *p*-value < 0.05. On the other hand, when searching for candidate genes for involvement in endosymbiosis, we raised the cutoff to fold change > 5 with a *p*-value < 0.05. Furthermore, because our study employed enough biological replicates (four samples per data point, each corresponding to a pool of three individuals) and a robust RNA-seq analysis pipeline, DESeq2, with adequate statistical treatment [75,76], RT-qPCR validation does not add much value [70]. Additionally, in our study, we used the RNA-seq data to contrast hypotheses (participation of the endosymbiont in the uric acid metabolism and in the synthesis of tyrosine for cuticle sclerotization) that have been well established in *B. germanica* for a long time [29,34]. In these pathways, several genes were differentially expressed, so even if 1.8% of the genes were not valid, the conclusion would not have changed. We also generated new hypotheses (candidate transporters and AMP-like factors for involvement in endosymbiosis), applying more restrictive conditions that will require subsequent verification and will be addressed in future works. Consequently, although RT-qPCR validation was not employed in this study, we are confident that this does not reduce the validity of our findings.

We detected around 800 transcripts differentially expressed between quasi-aposymbiotic and symbiotic fat bodies. As expected, they affected many different functions, but we consider that the most important for our study were those involved in (i) the metabolism of the aromatic amino acids phenylalanine and tyrosine, (ii) the metabolism of uric acid, (iii) the metabolite transport, and (iv) the innate immune system.

Thick and hard cuticles play essential roles in the survival of many insect species. In most of them, the synthesis and hardening of a new cuticle is required several times at each larval or nymphal molt and after the emergence of the adult at the final ecdysis. To produce the cuticle, several components have to be synthesized. They include cuticular proteins, chitin, and other essential compounds, such as catecholamine derivatives that act to produce melanin but, more importantly, to cross-link the cuticular proteins (sclerotization) [34]. To produce catecholamines, the availability of large amounts of tyrosine in concrete periods of the life cycle (at each molt or at the hardening of the adult cuticle) is crucial.

The role of symbionts in the production of tyrosine during insect development has been shown in several species, including many weevils, where an ancient endosymbiont (called *Nardonella*) has retained eight tyrosine biosynthesis genes even though it harbors one of the smallest bacterial genomes (slightly more than 200 kb) [11]. In cockroaches, tyrosine as well as phenylalanine are synthesized by *Blattabacterium* through a long pathway, in which chorismate is an intermediate for both amino acids [26]. An investigation of the origin of non-dietary phenylalanine in *P. americana* fed with a low-quality diet showed that the origins of this amino acid were bacterial (*Blattabacterium* and gut microbiota) and fungal (gut microbiota) [77]. Thus, in quasi-aposymbiotic individuals, both phenylalanine and tyrosine would be obtained from the diet and de novo from the gut microbiota. In fact, in our study, several genes encoding enzymes involved in the metabolism of phenylalanine and tyrosine were overexpressed in the fat body to produce large amounts of tyrosine for the synthesis of tyrosine-rich storage proteins (hexamerins) and as a precursor for the synthesis of dopamine, later converted into catecholamine cross-linkers involved in the sclerotization of the adult cuticle [34]. The very high expression of the hexamerins, especially in quasi-aposymbiotic adult females, reveals their importance. Hexamerins are synthesized in the fat body and released to the hemolymph, where they can be up to 50% of the total protein content [35].

We found in *B. germanica* quasi-aposymbiotic fat bodies that the genes for two enzymes of the synthesis of dopamine, *PAH* and *DDC,* were not only highly expressed but differentially overexpressed versus control individuals (Figure 3). However, the intermediate step catalyzed by TH was missing, leading us to wonder from where DDC obtains the L-DOPA substrate. The association of PAH and DDC in cuticle hardening and pigmentation has been reported in different insect species [78,79,80], and in *D. melanogaster*, the levels of dopamine increase in the critical periods for cuticle formation at each molt, pupariation, and adult ecdysis [81]. Various possible scenarios might explain the differential expression of the PAH and DDC genes and the absence of the expression of the TH gene in fat bodies. One of them would be that phenoloxidase with tyrosinase activity is involved in this step, although the encoding gene was not overexpressed. Another would be that *B. germanica* PAH is able to use tyrosine as an alternative substrate. This latter possibility was observed in the parasitic protist *Toxoplasma gondii*, which harbors two aromatic amino acid hydroxylase genes, encoding enzymes able to hydroxylase both phenylalanine [82]. On average, these enzymes are more similar to *B. germanica* PAH (51.3%) than to *B. germanica* TH (47.5 %) in the Biopterin-dependent aromatic amino acid hydroxylase domain (PFAM: PF00351).

Nitrogenous waste recycling (NWR) by host–symbiont interactions is an important way for insects to avoid nitrogen starvation. Nitrogenous wastes (e.g., uric acid, urea) are converted into ammonium that can be incorporated into organic compounds via the synthesis of glutamine by the host enzyme glutamine synthetase or into glutamate by the endosymbiont enzyme glutamate dehydrogenase [15,83]. The NWR pattern was identified in many Blattodea species, mainly through genomic or transcriptomic analysis. Depending on the species, the process takes place in the fat body, in the gut, or both [83]. In *B. germanica*, uric acid accumulated in urocytes is metabolized by the host and its endosymbiont *Blattabacterium* to produce essential and non-essential amino acids; the host degrades uric acid to the final product urea by the uricolytic pathway, and the endosymbiont degrades the urea with the urease activity generating ammonia [29]. In quasi-aposymbiotic individuals, the level of accumulated uric acid increases with time due to their inability to perform this metabolism [60].

Three enzymes in the uricolytic pathway were downregulated in quasi-aposymbionts (Figure 5). Urate oxidase was initially supposed to be the only enzyme responsible for the conversion of uric acid into allantoin in many species, including *B. germanica*, [29,84]. However, two genes sharing a common history of loss or gain events with urate oxidase were identified through phylogenetic genome comparison [85]. The two encoded proteins catalyze two consecutive steps following urate oxidation to 5-hydroxyisourate (HIU): hydrolysis of HIU to give 2-oxo-4-hydroxy-4-carboxy-5-ureidoimidazoline (OHCU) and decarboxylation of OHCU to give S-(+)-allantoin (Figure 5). The downregulation of the uric acid degradation pathway in the fat body of quasi-aposymbionts would explain the accumulation of urate deposits that were described in quasi-aposymbiotic cockroaches [60]. The excess of uric acid would be responsible of the downregulation of the pathways that in insects synthetize uric acid. Five enzymes that participate in the uric acid biosynthesis pathway, three involved in the synthesis de novo from amino acids from protein degradation, and two involved in the synthesis from purines obtained from nucleic acids turnover, are underexpressed in aposymbiotic fat bodies.

Establishing and maintaining endosymbiosis requires the metabolic integration between the host and the endosymbiont. To achieve this metabolic complementation, precise bidirectional movement of metabolites across the bacteriocyte membranes is needed, and this requires a specific repertoire of transporters. Some metabolites must be incorporated into the bacteriocyte from hemolymph (nutrients) or from adjacent cells (e.g., urea from urocytes) across the plasma membrane. Other metabolites must be imported into the symbiosome through the symbiosomal membrane to serve as nutrients or precursors for the synthesis of amino acids and vitamins by the endosymbiont. Finally, these biosynthetic products must be transported to the bacteriocyte cytoplasm through the symbiosomal membrane and subsequently exported to the hemolymph across the bacteriocyte plasma membrane [37]. Despite the importance of this molecular metabolic integration for the success of symbiosis, the precise molecular mechanisms that function at the host–endosymbiont interface remain largely unknown. No symbiosis-specific transporter has been identified in the *B. germanica-Blattabacterium* symbiotic system. Only in the *A. pisum-Buchnera* symbiotic system was a glutamine transporter identified that localizes to the bacteriocyte plasma membrane and allows the uptake of glutamine to the bacteriocyte from the hemolymph [86,87]. Furthermore, a non-essential amino acid transporter localized to the symbiosomal membrane that would allow bidirectional non-essential amino acid transfer between host and endosymbiont was also identified in this symbiotic system [88].

In this study, we identified membrane transporters underexpressed in quasi-aposymbionts that could be candidates for involvement in the transport of endosymbiosis-specific metabolites (Table 3). A urea transporter that localizes to the plasma membrane could be involved in the movement of urea across the urocyte or bacteriocyte plasma membranes, allowing the incorporation of urea into the cytoplasm of the bacteriocyte or across the symbiosomal membrane to supply urea to *Blattabacterium*. Two other underexpressed transporters, which could be involved in the transport of glutamate or phosphate, were also identified. Additionally, two trehalose transporters, as well as a zinc transporter, were found localized to the plasma membrane, suggesting that the import of trehalose and zinc to the bacteriocyte cytoplasm is essential for the maintenance of the endosymbiont. An analysis of gene expression in the bacteriocytes of the insect *Melophagus ovinus*, which contains the intracellular endosymbiont *Arsenophonus melophagy*, found that transporters of trehalose and zinc are also essential for endosymbiosis [89]. Finally, two underexpressed small membrane transporters were also identified as candidates for involvement in the endosymbiotic process. These transporters are formed by four TMSs, and their function is unknown. They could be localized to the symbiosomal membrane, participating in the transport of key metabolites for endosymbiosis, either into the vacuole or towards the cytoplasm of the bacteriocyte. Additional work is needed to elucidate the functional characteristics of these four TMS transporters in order to determine whether they play any fundamental role in the symbiosis in the *B. germanica-Blattabacterium* system.

The compartmentalization of symbionts in bacteriocytes is a strategy developed by many insect species to regulate the immune response in different body parts. Thus, the synthesis of AMPs may be reduced in bacteriocytes, while the immune response may be high and inducible in other tissues [90]. Examples are the insect–bacterial mutualistic relationships of the hymenopteran *Camponotus floridanus* with *Blochmannia floridanus* [91], the coleopteran *Sitophilus* spp. with *Sodalis pierantonius* [46,92], and the dipteran *Glossina morsitans* with *Wigglesworthia glossinidia* [62]. This type of tissue-specific expression was also demonstrated for exosymbionts, such as the dipteran *Bactrocera dorsalis* and gut symbionts Enterobacteriaceae [93] and the hemipteran *Riptortus pedestris* and its gut symbionts of the genus *Burkholderia* [94]. On the other hand, the expression of a specific AMP possibly evolved to control the endosymbiont population, as is the case with coleoptericin A in the genus *Sitophilus* [46,51].

The key genes expressed in bacteriocytes involved in avoiding the induction of the immune signaling pathways encode PGRPs with amidase activity, which will be able to degrade the peptidoglycan molecules (Lys-type peptidoglycan of Gram-positive bacteria and DAP-type peptidoglycan of Gram-negative bacteria) that would otherwise induce the expression of the AMP genes [90]. In *D. melanogaster*, the enzymatic amidase activity was proposed in several genes, such as *PGRP-LB* [95] or *PGRP-SC1B* [96] (see [97] for a summary). PGRP-LB and other PGRP proteins with amidase activity provide protection for symbiotic bacteria by binding to and cleaving peptidoglycan molecules derived from these bacteria, making them invisible to the immune system. In *B. dorsalis*, PGRP-LB and PGRP-SB restrain immune effector expression in the midgut to establish protective zones for symbiotic bacteria [93]. The presence of the symbionts activates the expression of these PGRP genes, something that can be observed from their effects after antibiotic treatment [93]. A similar role of PGRP-LB in bacteriocytes to prevent antibacterial immune cascades, which can damage the endosymbiont population, was reported in *G. morsitans* [62] and *Sitophilus zeamais* [92].

The domain analysis carried out in this study predicted the amidase catalytic domain in PGRP-LB_1 (both isoforms), PGRP-LB_2, and the PGRP-LA-like. However, while the first two of these showed the four conserved residues involved in the Zn^2+^ interaction in *Drosophila* PGRP-LB (H42, Y78, H152, and C160, accession: Q8INK6) required for enzymatic activity [98], the PGRP-LA-like displays differences in two of them, suggesting that it has no amidase activity. Some residues responsible for the DAP/Lys specificity were also conserved [98] (Figure S3). The fact that the *PGRP-LB_1* gene was underexpressed in quasi-aposymbionts suggests that in symbiotic bacteriocytes, the presence of a large population of the endosymbiont induces the expression of the intracellular isoform of PGRP-LB_1 (DN3036_c0_g1_i2), avoiding the stimulation of the IMD pathway. This is probably the reason why the level of expression of AMP genes in *B. germanica* is low compared with hemolymph (hemocytes) and salivary glands, two important locations for controlling pathogens [50]. The predicted cell membrane PGRP-LA-like protein was also underexpressed in quasi-aposymbionts, but its function is unknown.

In some insect–bacterial symbiotic relationships, specific AMPs evolved to control the population of the bacterial symbiont. In weevils of the genus *Sitophilus*, coleoptericin A operates within the bacteriocytes and controls symbiont cell division [46]. In addition, the *Sitophilus colA* gene transcript level exhibits a different profile from the other AMP genes, increasing in parallel with endosymbiont charge and being non-detected in midguts isolated from aposymbiotic individuals [99]. In the *A. pisum-Buchnera* symbiosis, a novel class of genes encoding bacteriocyte-specific cysteine-rich (BCR) small proteins with signal peptides that are overexpressed in bacteriocytes was identified [53]. BCR family genes possibly evolved from defensin-type AMPs. They are restricted to the aphid lineage, and some members show antimicrobial properties in vitro, even affecting membrane permeability [54,55]. Thus, it was proposed that BCR proteins are AMP-like factors that may be used in the control of *Buchnera* growth or as facilitators for metabolite exchange between *Buchnera* and host cells [54].

We expected to detect some AMP genes underexpressed in quasi-aposymbionts, which could be related to the control of the *Blattabacterium* population in the bacteriocyte cells. We detected three genes (*defensin_g9*, *defensin_g10*, and *termicin_g4*) that fulfilled this starting criterion. However, the fact that their main site of expression in control individuals was the hemolymph, with a level at least two orders of magnitude higher, leads us to consider that they are probably not related to this task. We then searched for underexpressed genes encoding small proteins with some amino acid composition characteristics typical of AMPs, trying to identify potential non-canonical AMPs that could be involved in endosymbiont control and bacteriocyte homeostasis.

We identified several candidate genes for encoding AMP-like factors involved in the endosymbiosis in *B. germanica*. For this purpose, we selected underexpressed transcripts that encode proteins smaller than 200 amino acids and are rich in cysteine (Table 5). Among these, five were soluble proteins with extracellular localization, of which four contained signal peptides indicating that they could be exported to the symbiosome. Three transcripts encode proteins that show homology to proteins related to the immune system: one (encoded by DN2464_c0_g1_i3) has homology to type c-1 lysozymes, and the other two display homology to C-type lectins (encoded by DN48391_c0_g1_i1 and DN17653_c0_g1_i2).

Regarding lysozymes, they play an important role in the immune system of insects, displaying antimicrobial activity by lysing cell wall peptidoglycan and disrupting bacterial membranes, although a role in symbiosis was also proposed for these proteins. In the *A. pisum-Buchnera* symbiosis, transcriptomic analysis showed that genes encoding putative lysozymes represented the genes with the highest expression rate in the pea aphid bacteriocyte. From this result, lysozymes were proposed as candidate molecules involved in the control and maintenance of the endosymbiont population, probably triggered by the lysosomal breakdown of *Buchnera* [100,101]. However, in other symbiotic systems, lysozyme genes are downregulated if the endosymbiont is present. In the bean bug *R. pedestris*, which harbors the extracellular bacterial symbiont *Burkholderia* in crypts in the midgut, a lysozyme gene is upregulated in aposymbiotic relative to symbiotic individuals [102], and in the grain weevils *S. zeamais* and *S. oryzae*, the expression of lysozyme genes is downregulated in the bacteriocytes harboring *Sodalis* endosymbiotic bacteria [103].

C-type lectins, like the one we detected, are calcium-dependent carbohydrate-binding proteins that play an important role in insect innate immune response, as well as in maintaining gut microbiome homeostasis. They are a type of pattern-recognition receptor (in addition to the PGRPs family already discussed) that can identify pathogen-associated molecular patterns on the surface of bacteria by carbohydrate-binding interactions. Some lectins have antimicrobial properties: they are secreted and able to kill bacteria by affecting their membrane. Moreover, secreted lectins were related to symbiosis due to their ability to recognize symbionts by carbohydrate-binding interactions. In the cnidarian-*Symbiodinium* symbiosis, lectins mediate the recognition and internalization of endosymbionts, reduce the rate of cell division regulating *Symbiodinium* density, and modulate the host immune response [104,105]. In the amoeba *Dictyostelium discoideum*, endosymbiosis is induced by a secreted lectin that binds bacteria, protecting them from extracellular killing [106]. Finally, in the mosquito *Aedes aegypti*, lectins facilitate gut colonization by the commensal microbiome by coating the bacterial surface, counteracting the activity of AMPs [107].

The other two selected candidates correspond to transcripts that encode small orphan proteins with a high cysteine content presenting no homology to other proteins documented in the database (DN3965_c0_g1_i1 and DN5401_c0_g2_i1), meaning that they could encode lineage-specific proteins. Both proteins would have extracellular localization, although only the protein encoded by DN3965_c0_g1_i1 contains a signal peptide. These proteins are AMP-like factor candidates that could possibly be involved in the endosymbiosis control and homeostasis of the bacteriocyte.

Future work will determine whether the expression in the fat body of these identified genes (PGRPs, transporter proteins, and AMP-like factors) is localized in the bacteriocyte and, more specifically, in the symbiosomal vacuole, as well as identify the roles they may have in the endosymbiosis in *B. germanica*.

In conclusion, the comparison of quasi-aposymbiotic and control individuals provided clues on the genes involved in protecting the symbiont population and the host–symbiont relationship. We also determined that the loss of the metabolism of the endosymbionts could be partially compensated by the regulation of host gene expression to favor the synthesis of tyrosine or to reduce the metabolism of uric acid to urea.

## 4. Materials and Methods

### 4.1. Insect Rearing

A population of *B. germanica* was raised in plastic containers at 25 °C and a relative humidity of 60% in 12/12 h light/dark cycles in climatic chambers at the Institute for Integrative Systems Biology (University of Valencia-CSIC). The diet was based on dog food (Teklad Global 21% protein dog diet, 2021C, Envigo, Madison, WI, USA) and water provided ad libitum. When needed, rifampicin (Alfa Aesar, Kandel, Germany) was provided with the water at 0.1 mg/mL, and feces from a control population were added to the diet during rifampicin treatment.

To perform the analyses, a *B. germanica* quasi-aposymbiotic population (in which the endosymbiont *Blattabacterium* population was greatly reduced) was generated as follows: synchronized adults (0−48 h after emergence from nymphs) at first generation were treated with the antibiotic rifampicin (0.1 mg/mL) until ootheca hatching to form the next generation as published previously [59]. Nymphs of the second generation reached adulthood without additional contact with the antibiotic. Ten-day-old adult females from the quasi-aposymbiotic population and from a control population (not treated with the antibiotic) were dissected, and fat bodies were extracted. For both populations, adult individuals were collected from different containers to avoid population bias, and only females were selected to avoid bias due to sexual dimorphism.

### 4.2. Insect Dissection and Collection of Samples

Cockroaches were sedated with CO_2_, and sequentially cleaned with 10% bleach, 70% ethanol, and double-washed with Type II water. Each cockroach was fixed with entomological needles in supine position on a silicone plate, and the fat body was collected. Four replicates (each one corresponding to a pool of three individuals) were obtained for each population. Samples were collected in previously cooled Eppendorf tubes, treated overnight with RNAlater (Invitrogen, Waltham, MA, USA), which was then removed, and stored at −80 °C until RNA extraction.

### 4.3. RNA/DNA Extraction and Sequencing

RNA and DNA were extracted from fat body with the NucleoSpin RNA/DNA Buffer Set kit (Macherey-Nagel, Düren, Germany) according to the manufacturer’s recommended instructions. RNA and DNA were quantified with Nanodrop and Qubit. The endosymbiont load was determined by qPCR using the DNA of each individual, following the protocol previously described [57]. In this way, we confirmed that the endosymbiont load of quasi-aposymbiont individuals decreased by at least 5 orders of magnitude (Table S2). RNA sequencing was carried out at the *Servei Central de Suport a la Investigació Experimental* (SCSIE, University of Valencia). RNA integrity and quality, as well as mRNA enrichment, were analyzed before proceeding with library preparation. Sequencing was performed using Illumina NextSeq 550 to produce around 5.5 million reads per sample (paired-end reads of 2 × 150 nucleotides).

### 4.4. Read Filtering, Transcriptome Assembly and Expression Analysis

The quality of reads was checked with FastQC v0.11.9 (http://www.bioinformatics.babraham.ac.uk/projects/fastqc/, accessed on 12 September 2022). Correction of random sequencing errors in Illumina RNA-seq reads was performed with Rcorrector v1.0.3 (https://usegalaxy.eu/, accessed on 26 October 2022) [108]. Trimmomatic v0.38.1, with default parameters (https://usegalaxy.eu/, accessed on 26 October 2022) [109], was used to remove residual adapters that may not have been removed in the standard sequencing protocol.

Prior to the expression analyses, we decided to map the reads on a de novo transcriptome assembly of *B. germanica* 10-day-old adult females instead of the coding genes annotated in the genome [66]. The reason for this was that we observed, in previous studies, that a relevant number of genes involved in immunity were not annotated in the genome, especially those of small length, such as AMP genes [49,50]. To increase the diversity and coverage of the de novo transcriptome assembly, reads from 24 additional samples of 2 other tissues (hindgut and salivary glands) from *B. germanica* 10-day-old adult females available in our laboratory were added to the eight fat body samples as the input for the assembly. Trinity v2.11.0 [110] was used to perform de novo assembly with paired-end reads from the 32 samples. Data processing was carried out on a CentOS v7.9.2009 server. A total of 210,777 transcripts were obtained. Because the number of transcripts was very high, quantification was performed by pseudoalignment with Kallisto v0.46.2 [111]. The program pseudoaligned 117.9 million reads with an average of 155.5 nucleotides. Transcripts with very low expression (less than 50 counts or with a TPM value smaller than 1) were removed. This produced a transcriptome with 37,239 transcripts. Assembled transcripts corresponding to AMP genes and IMD pathway genes were replaced by those previously characterized [49,50], producing a final transcriptome of 37,249 transcripts.

A Kallisto index was obtained with this transcriptome. The abundance of each transcript in the four control and the four quasi-aposymbiotic fat body samples was estimated with Kallisto [111]. The R package DESeq2 1.36.0 [75] with standard parameters using the quantification values from Kallisto was used to call for differentially expressed transcripts. Transcripts were considered differentially expressed if the absolute value of log2FoldChange was higher than 1 (fold change > 2 for overexpressed and underexpressed) and with the adjusted *p*-value cutoff set to 0.05.

### 4.5. Functional Annotation

To identify the protein-coding of differentially expressed transcripts, TransDecoder v5.5.0 [102] with a minimum length of 60 codons was used. Predicted proteins (in some transcripts, more than one hypothetical protein was predicted) were annotated with eggNOG-mapper 2.1.9 with default conditions [112]. The proteins without eggNOG functional assignments were used as queries in a BLASTP against the non-redundant protein sequences (nr) database from NCBI (E-value cutoff 10^−5^). Both types of annotations, including expression values, transcripts, and protein lengths, can be found in Table S1.

KEGG pathway map numbers annotated by eggNOG were used to group transcripts for Figure 1 and Figure 2. A few transcripts without KEGG map annotations but with KO numbers were rescued using KEGG mapper tool (https://www.genome.jp/kegg/mapper/, accessed on 11 November 2022).

### 4.6. Signal Prediction and Subcellular Localization

The prediction of eukaryotic protein subcellular localization using deep learning was performed with DeepLoc 2.0 (https://services.healthtech.dtu.dk/services/DeepLoc-2.0/, accessed on 16 October 2023) using the High-quality model. Signal peptides were predicted with SignalP 6.0 (https://services.healthtech.dtu.dk/services/SignalP-6.0/, accessed on 16 October 2023). Other protein features were predicted with InterProscan (https://www.ebi.ac.uk/interpro/search/sequence/, accessed on 16 October 2023). To determine the presence of transmembrane segments (TMSs), DeepTMHMM (https://dtu.biolib.com/DeepTMHMM, accessed on 16 October 2023) was employed. To predict membrane protein functions, BLASTP analysis was performed against the Transporter Classification Database (TCDB; http://www.tcdb.org, accessed on 16 October 2023) [113].

### 4.7. Phylogenetic Analysis

Protein alignments, best evolutionary model predictions, and phylogenetic reconstructions were performed with several methods implemented in MEGA v11.0.13 [114]. Multiple sequence alignments were performed with MUSCLE. For phenoloxidases, hemocyanins, and hexamerins (Figure 4), a phylogenetic reconstruction was inferred using the Neighbor-Joining method. This analysis involved 39 sequences in a multiple alignment of 456 amino acid sites. Evolutionary distances were calculated using the JTT matrix-based method, which was the best-fitting substitution model estimated by MEGA. The complete deletion option was used for gap treatment. It means that sites containing a gap in at least one sequence are removed from the alignment prior to inferring the phylogeny. A bootstrap test (1000 replicates) was performed. For the PGRP proteins (Figure 6), a phylogenetic reconstruction was inferred using the maximum likelihood method. This analysis involved 50 sequences in a multiple alignment of 178 amino acid sites, including most of the IPR036505 domain superfamily. Due to the short length of the alignment, the “All sites” option was used to handle gaps. The Le_Gascuel_2008 model with discrete Gamma distribution (LG + G) was used, which was the best-fitting substitution model estimated by MEGA. The tree with the highest log likelihood (−12,697.81) is shown. A bootstrap test (1000 replicates) was performed.

## Figures and Tables

**Table 1:** Classification of transcripts overexpressed and underexpressed in quasi-aposymbiotic fat bodies.

Type of Transcript	Over	Under
Coding transcripts with annotation in eggnog	160	227
Coding transcripts with a BLASTP hit in NCBI but without eggNOG annotation	27	55
Coding transcripts without either annotation in eggNOG or hit in NCBI	59	60
Non-coding transcripts	113	105
Transcripts from *Blattabacterium* genome	0	13
TOTAL	359	460

**Table 2:** Transcripts overexpressed in quasi-aposymbiotic fat bodies that were potentially involved in cuticle sclerotization. Abbreviations: TPM (transcripts per million); q-Apo (quasi-aposymbiont); aa (amino acids).

Transcript ^1^	Fold Change ^2^	TPM q-Apo	TPM Control	Annotation from UniProt or Modified	ENA Accession Equivalency	TransDecoder Length (aa)	TransDecoder Information
DN206_c21_g1_i1	6.5	750.5	91.2	Phenylalanine 4-monooxygenase	PSN42902.1	453	complete
DN25_c0_g1_i4	4.2	42.7	7.7	Aminotran_1_2 domain-containing protein	PSN48874.1	361	complete
DN37368_c0_g1_i1	15.1	166.3	9.3	Aromatic-L-amino acid decarboxylase	PSN57389.1	480	complete
DN5435_c0_g1_i1	4	69.9	11.2	y(+)L-type amino acid transporter 2	PSN41882.1	495	complete
DN85219_c0_g1_i1	18.3	10	0.4	Cuticular protein RR2 type	PSN41655.1	154	complete
DN8606_c0_g1_i2	7.7	14	1.3	Endochitinase	PSN50302.1	431	complete
DN15_c0_g1_i2	32.9	2452.8	54.3	Storage protein/ hexamerin	PSN36665.1	626	3prime_partial
DN15_c0_g1_i4	22.7	5041.9	159.6	Storage protein/ hexamerin	PSN36665.1	676	complete
DN15_c0_g1_i6	5.8	15,488.8	1797.4	Storage protein/ hexamerin	Absent	676	complete

^1^ For simplicity, “TRINITY_” has been removed at the start of the transcript names. ^2^ Fold change (quasi-aposymbiont/control fat bodies) obtained from DeSeq2 analysis.

**Table 3:** Transcripts underexpressed more than five-fold in quasi-aposymbiotic fat bodies encoding proteins with more than three TMSs (according to DeepTMHMM analysis) and membrane localization (according to DeepLoc analysis). Domains were identified with InterProScan and cellular localization was analyzed with DeepLoc. Abbreviations: TPM (transcripts per million); q-Apo (quasi-aposymbiont); TMSs no.: number of transmembrane segments predicted with DeepTMHMM; TCDB family homology: indicates the name and TC number of the transporter family from the TCDB database with which it shows similarity according to BLASTP analysis. Putative functions were deduced after the best BLASTP hits against the TCDB.

Transcript ^1^	Domain	Fold Change	TPM Mean q-Apo	TPM Mean Control	DeepLoc Localizations	TMSs No.	TCDB Family Homology	Putative Functions
DN12960_c0_g1_i2	PF07690	20.9	0.8	11.5	Cell membrane	12	Anion:Cation Symporter Family (TC# 2.A.1.14)	Glutamate or phosphate transporter
DN1327_c1_g1_i8	PF00083	12.9	3.4	35.7	Cell membrane	12	Sugar Porter Family (TC# 2.A.1.1)	Trehalose transporter
DN1382_c0_g1_i1	PF02535	10.4	2.1	18.5	Cell membrane| Lysosome/ Vacuole	8	Zinc (Zn^2+^)-Iron (Fe^2+^) Permease Family (TC# 2.A.5)	Zinc transporter
DN2082_c0_g1_i1	PF03253	15.5	0.3	3	Cell membrane| Lysosome/ Vacuole	10	Urea Transporter Family (TC# 1.A.28)	Urea transporter
DN22022_c0_g2_i1	PF12832	19.6	0.7	9.5	Cell membrane	12	Unidentified Major Facilitator-14 Family (TC# 2.A.1.65)	Unknown
DN2708_c1_g1_i2	PF00083	8.7	4.2	29.5	Cell membrane	12	Sugar Porter Family (TC# 2.A.1.1)	Trehalose transporter
DN3532_c0_g1_i1	PF00023	11.5	2.3	18.5	Cell membrane	6	Transient Receptor Potential Ca^2+^/Cation Channel Family (TC# 1.A.4)	Ca^2+^/cation channel
DN3824_c0_g1_i2	PF01080	10.6	0.9	6.7	Cell membrane| Endoplasmic reticulum|Golgi apparatus	9	Presenilin Endoplasmic Reticulum Ca^2+^ Leak Channel Family (TC# 1.A.54)	Ca^2+^ channel
DN4893_c0_g1_i1	PF07690	5.9	4.5	17.3	Cell membrane	11	Anion:Cation Symporter Family (TC# 2.A.1.14)	Glutamate or phosphate transporter
DN74365_c0_g1_i1	PTHR34609	6.8	2.8	13.3	Lysosome/ Vacuole	4	4 TMS Multidrug Endosomal Transporter Family (TC# 2.A.74)	Unknown
DN8_c1_g1_i10	PTHR34609	22.3	4.1	68.9	Lysosome/ Vacuole	4	4 TMS Multidrug Endosomal Transporter Family (TC# 2.A.74)	Unknown
DN82467_c0_g1_i1	PF05978	7.2	19.7	96	Cell membrane	12	N-Acetylglucosamine Transporter Family (TC# 2.A.1.58)	Potassium channel regulatory protein
DN9218_c0_g1_i4	PF00005	7	3.9	18.5	Cell membrane| Endoplasmic reticulum	6	Eye Pigment Precursor Transporter Family (TC# 3.A.1.204)	ABC transporter

^1^ For simplicity, “TRINITY_” was removed at the start of the transcript names.

**Table 4:** Genes involved in Toll-Imd pathways underexpressed in quasi-aposymbiotic fat bodies. Fold change (expression in control/expression in quasi-aposymbionts). Abbreviations: TPM (transcripts per million); q-Apo (quasi-aposymbiont); aa (amino acids).

Gene Name	Transcript ^1^	Protein Length (aa)	Fold Change	TPM Mean q-Apo	TPM Mean Control	Localization ^2^	Signal Peptide ^2^
*cactus*	DN1475_c0_g1_i1	438	5	20.1	76.7	Cytoplasm	
*cactus*	DN1475_c0_g1_i2	440	5.6	11.3	43.9	Cytoplasm	
*cactus*	DN1475_c0_g1_i4	456	1319.5	0	31.3	Cytoplasm	
*pelle*	DN733_c0_g1_i4	728	2.3	10.7	17.5	Cytoplasm	
*PGRP-LA-like*	DN3159_c1_g1_i1	226	3.9	18.3	49.1	Cell membrane	Yes
*PGRP-LB_1*	DN3036_c0_g1_i2	182	5.5	8	30.1	Cytoplasm| Extracellular	
^3^ *defensin_g9*	defensin_g9	71	7	36.9	162.2	Extracellular	Yes
^3^ *defensin_g10*	defensin_g10	71	8.5	31.9	165.7	Extracellular	Yes
^3^ *termicin_g4*	DN4880_c0_g1_i1	61	7.9	53.8	262.2	Extracellular	Yes
*PGRP-LB_1*	^4^ DN3036_c0_g1_i1	237	-	1.3	0	Extracellular	Yes
*PGRP-LB_2*	^4^ DN4891_c0_g1_i1	191	-	5.5	7	Extracellular	Yes

^1^ For simplicity, “TRINITY_” was removed at the start of the transcript names. ^2^ DeepLoc predicted the subcellular localization. Signal peptides were predicted with SignalP. ^3^ The names of the genes *defensin_g9* and *defensin_g10* were previously described [49], while *termicin_g4* is a new termicin gene detected in the present study. ^4^ These transcripts were not differentially expressed but are included because they come from PGRP-LB genes.

**Table 5:** Transcripts underexpressed more than five-fold in quasi-aposymbiotic fat bodies encoding cysteine-rich proteins smaller than 200 amino acids. Domains starting with PF and PTHR belong to PFAM and Panther databases, respectively. Domains and signal peptides were identified with InterProScan. Abbreviations: Prot. length (protein length); aa (amino acids); Cys no. (number of cysteines); SignalP: signal peptide; TPM (transcripts per million); q-Apo (quasi-aposymbiont); TMSs no.: number of transmembrane segments predicted with DeepTMHMM.

Transcript ^1^	Protein Length (aa)	Cys No.	Domain	SignalP	Fold Change	TPM Mean q-Apo	TPM Mean Control	DeepLoc Localizations	TMSs No.	AMP-Like Factor Candidate
DN12569_c0_g1_i1	127	14	PF00008	Yes	6.8	7.5	31.9	Extracellular		No
DN17653_c0_g1_i2	186	9	PF00059	Yes	11	1	7.8	Extracellular		Yes
DN2464_c0_g1_i3	140	8	PF00062	Yes	10.3	2.1	13.9	Extracellular		Yes
DN3965_c0_g1_i1	108	15		Yes	7.1	3.5	17.5	Extracellular		Yes
DN43375_c0_g1_i1	106	8			14.3	1.7	16.2	Mitochondrion		No
DN48391_c0_g1_i1	188	10	PF00059	Yes	12.2	0.8	7.7	Extracellular		Yes
^2^ DN4880_c0_g1_i1 termicin_g4	61	7	PF11415	Yes	7.9	53.8	262.2	Extracellular		No
DN5401_c0_g2_i1	67	7			47.1	0.2	9.3	Extracellular	1	Yes
DN54153_c0_g1_i1	102	6			22.2	0.7	10.1	Cytoplasm| Nucleus		No
DN7034_c0_g1_i1	150	11	PF17064	Yes	6.3	5.9	24.2	Cell membrane		No
DN74365_c0_g1_i1	195	6	PTHR34609		6.8	2.8	13.3	Lysosome/ Vacuole	4	No
DN8_c1_g1_i10	174	10	PTHR34609		22.3	4.1	68.9	Lysosome/ Vacuole	4	No
^2^ defensin_g10	71	7	PF01097	Yes	8.5	31.9	165.7	Extracellular		No
^2^ defensin_g9	71	7	PF01097	Yes	7	36.9	162.2	Extracellular		No

^1^ For simplicity, “TRINITY_” was removed at the start of the transcript names. ^2^ The names of the genes *defensin_g9* and *defensin_g10* were previously described [49], while *termicin_g4* is a new termicin gene detected in this study.

## Data Availability

The data for this study have been deposited in the European Nucleotide Archive (ENA) at EMBL-EBI under accession number PRJEB67739 (ERS16510948-ERS16510955). The assembled sequences are available upon request.

## Supplementary Materials

The following supporting information can be downloaded at: https://www.mdpi.com/article/10.3390/ijms25084228/s1, Figure S1: Phylogenetic analysis of DOPA decarboxylase and related proteins in several insects; Figure S2. Normalized expression (GeTMM) of the gene encoding the termicin_g4 protein in seven sample types of *B. germanica*; Figure S3: Alignment of the PGRP domain superfamily (IPR036505) in several insect proteins; Table S1: Analyses of transcripts over- and underexpressed in quasi-aposymbiotic fat bodies; Table S2: Endosymbiont load of pooled quasi-aposymbiont and control fat bodies determined by qPCR.
