# Supplementary material for: Comparative Transcriptomics of Fat Bodies between Symbiotic and Quasi-Aposymbiotic Adult Females of Blattella germanica with Emphasis on the Metabolic Integration with Its Endosymbiont Blattabacterium and Its Immune System
Source: Int J Mol Sci. 2024 Apr 11;25(8):4228. doi: 10.3390/ijms25084228 (PMC11050582; doi:10.3390/ijms25084228)
Supplement: Supplementary file 1 [file ijms-25-04228-s001.zip › Figure S1.pdf]

Comparative Transcriptomics of Fat Bodies Between Symbiotic and Quasi-Aposymbiotic Adult Females of *Blattella germanica* with Emphasis on the Metabolic Integration with its Endosymbiont *Blattabacterium* and Its Immune System

Francisco J. Silva, Rebeca Domínguez-Santos, Amparo Latorre and Carlos García-Ferris

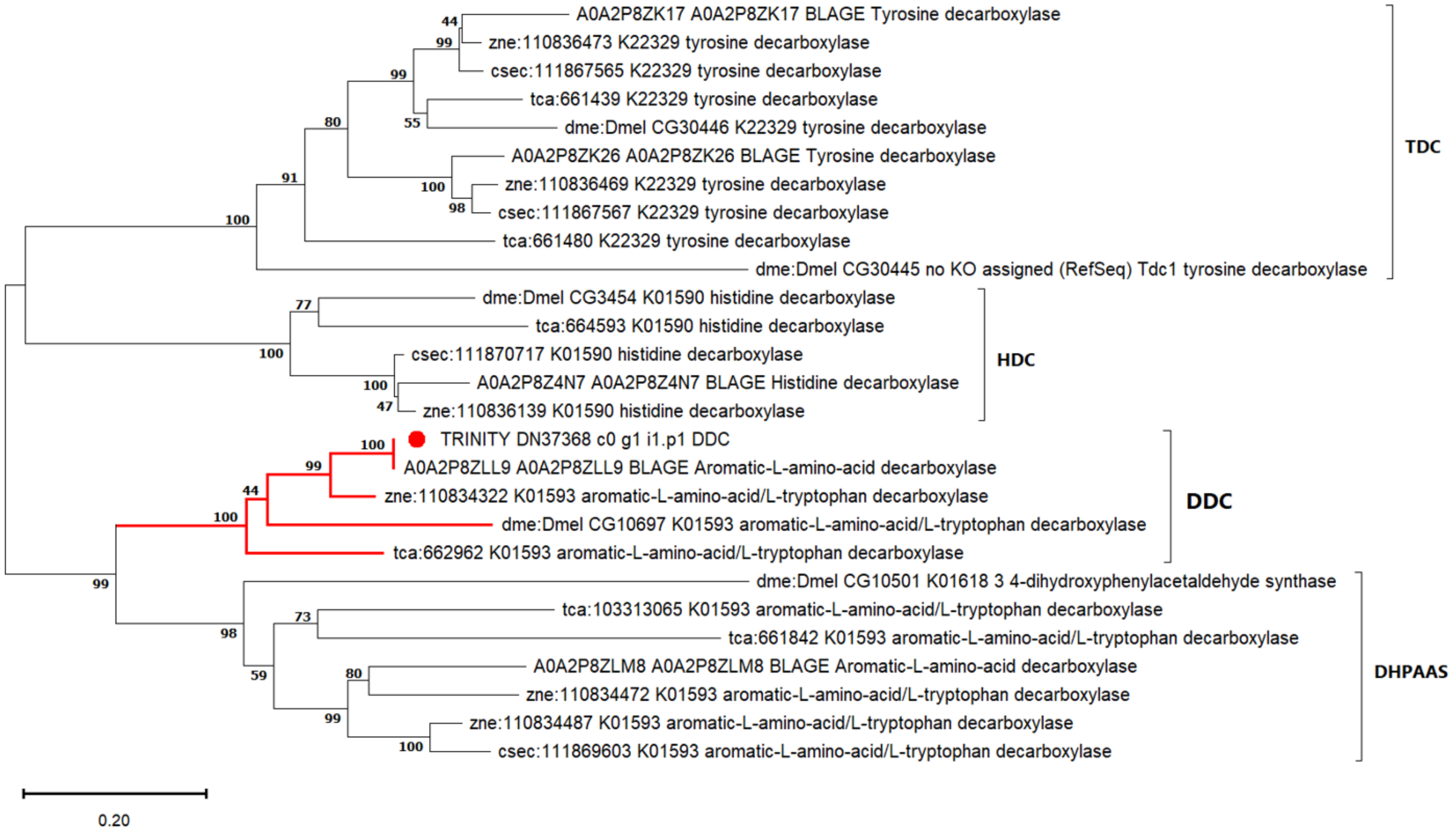

Figure S1. Phylogenetic analysis of DOPA decarboxylase and related proteins in several insects. Phylogenetic reconstruction of tyrosine decarboxylase (TDC), histidine decarboxylase (HDC), DOPA decarboxylase (DDC) and 3,4-dihydroxylphenylacetaldehyde synthase (DHPAAS) in several insects. Proteins were selected from KEGG genes, except those of *B. germanica* (Bger) obtained from Uniprot and the protein encoded by TRINITY_DN37368_c0_g1. Abbreviated taxonomic codes: csec (*Cryptotermes secundus*, drywood termite), dme (*Drosophila melanogaster*, fruit fly), tca (*Tribolium castaneum*, red flour beetle), and zne (*Zootermopsis nevadensis*, dampwood termite). Uniprot proteins from *B. germanica* include the code BLAGE. The phylogenetic tree was inferred with the Neighbor-Joining method, the JTT + G evolutionary model and 1000 bootstrap replicates on an amino acid alignment of 399 residues with the complete deletion option. The scale bar corresponds to 0.20 estimated amino acid substitutions per site. DDC clade is marked in red. A red circle marks the location of the analyzed *B. germanica* decarboxylase protein.
