# Supplementary material for: Comparative Transcriptomics of Fat Bodies between Symbiotic and Quasi-Aposymbiotic Adult Females of Blattella germanica with Emphasis on the Metabolic Integration with Its Endosymbiont Blattabacterium and Its Immune System
Source: Int J Mol Sci. 2024 Apr 11;25(8):4228. doi: 10.3390/ijms25084228 (PMC11050582; doi:10.3390/ijms25084228)
Supplement: Supplementary file 1 [file ijms-25-04228-s001.zip › Figure S2.pdf]

Comparative Transcriptomics of Fat Bodies Between Symbiotic and Quasi-Aposymbiotic Adult Females of *Blattella germanica* with Emphasis on the Metabolic Integration with its Endosymbiont *Blattabacterium* and Its Immune System

Francisco J. Silva, Rebeca Domínguez-Santos, Amparo Latorre and Carlos García-Ferris

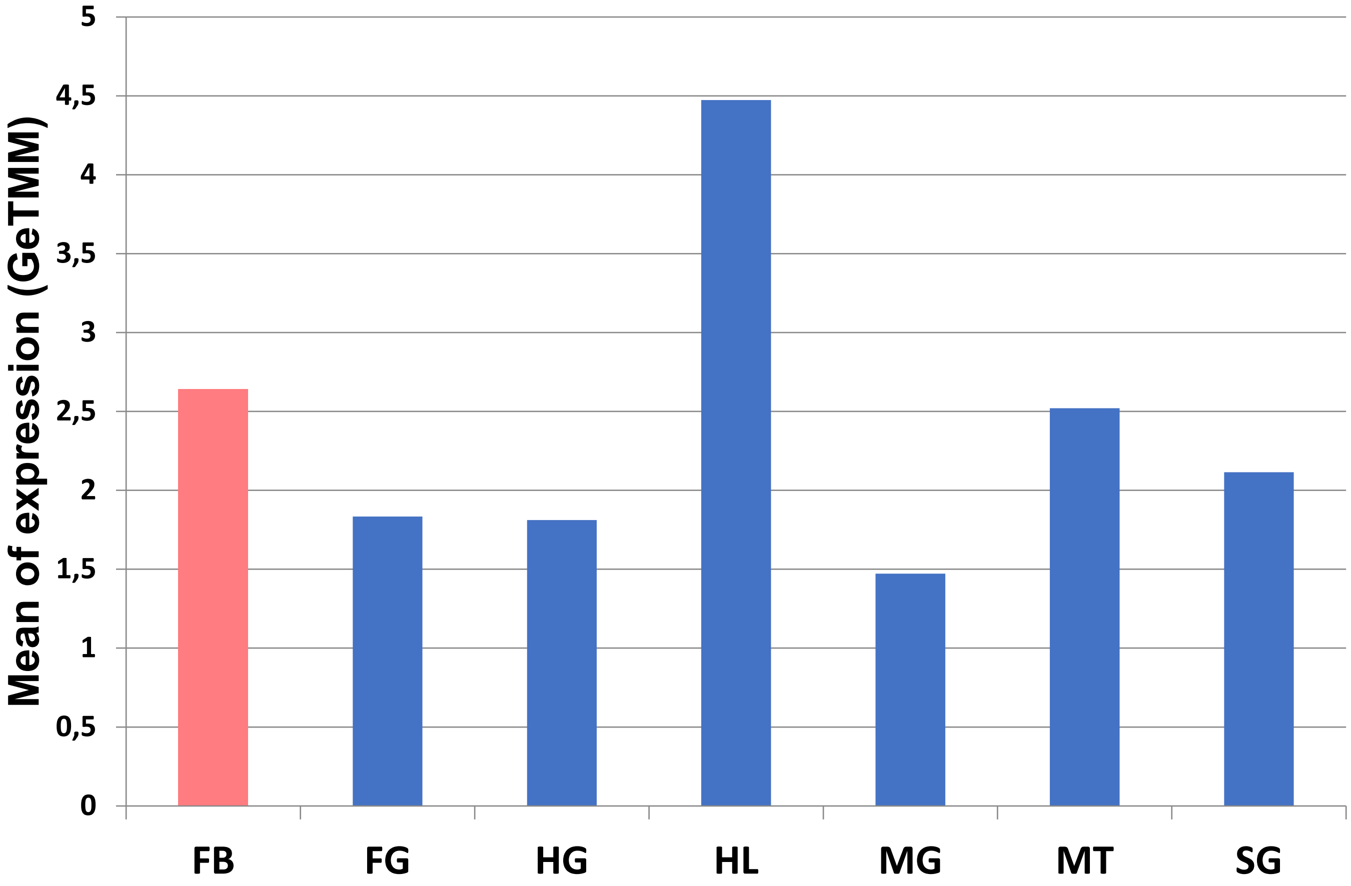

Figure S2. Normalized expression (GeTMM) of the gene encoding the termicin_g4 protein in seven sample types of *B. germanica*. Values are the means of expression of four replicates in the six tissues and three in hemolymph. Abbreviations: FB (fat body), FG (foregut), HG (hindgut), HL (hemolymph), MG (midgut), MT (Malpighian tubules) and SG (salivary glands). Y-axis is in log scale. Data were obtained from Zuber, L.; Domínguez-Santos, R.; García-Ferris, C.; Silva, F.J. Identification of the gene repertoire of the IMD pathway and expression of antimicrobial peptide genes in several tissues and hemolymph of the cockroach *Blattella germanica*. *Int. J. Mol. Sci*. **2022**, *23*, 8444. https://doi.org/10.3390/ijms23158444.
