# Supplementary material for: Comparative Transcriptomics of Fat Bodies between Symbiotic and Quasi-Aposymbiotic Adult Females of Blattella germanica with Emphasis on the Metabolic Integration with Its Endosymbiont Blattabacterium and Its Immune System
Source: Int J Mol Sci. 2024 Apr 11;25(8):4228. doi: 10.3390/ijms25084228 (PMC11050582; doi:10.3390/ijms25084228)
Supplement: Supplementary file 1 [file ijms-25-04228-s001.zip › Figure S3.pdf]

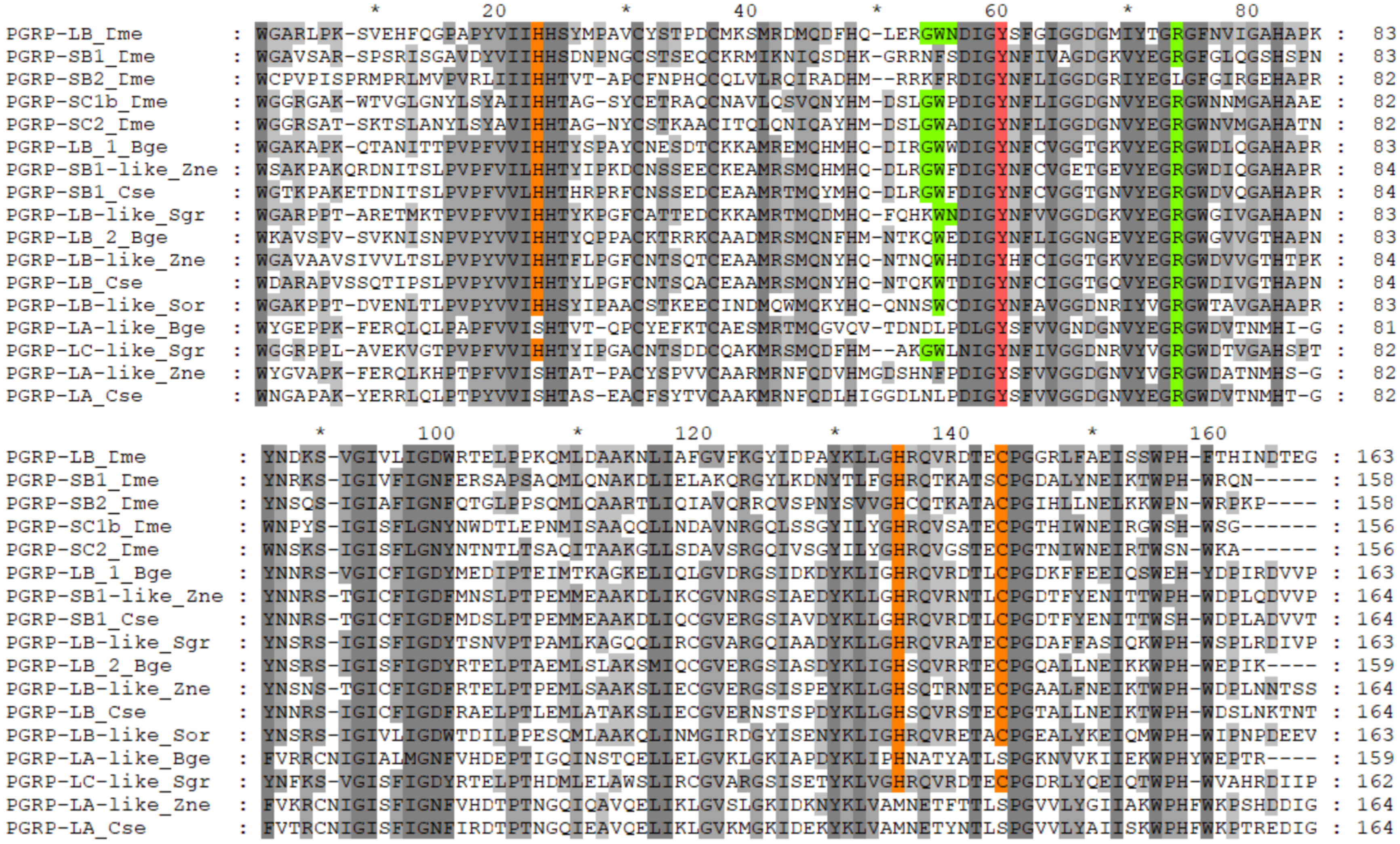

Figure S3. Alignment of the PGRP domain superfamily (IPR036505) in several insect proteins. The alignment includes those proteins whose branches were highlighted in colors in the phylogeny of Figure 6, with the addition of several other PGRP proteins, such as *D. melanogaster* PGRP-LB and other *D. melanogaster* PGRP proteins with amidase activity. The residues highlighted in orange are responsible for the Zn^{2+} interaction in *D. melanogaster* PGRP-LB. The residue highlighted in red makes a fourth coordination with Zn^{2+} through a water molecule in *Drosophila* PGRPLB. Those residues highlighted in green are responsible for the DAP/Lys specificity. The alignment range was from W38 to G200 of *D. melanogaster* PGRP-LB (NP_731575.1). Abbreviations: Dme (*D. melanogaster*), Bge (B. germanica) Zne (*Zootermopsis nevadensis*), Cse (*Cryptotermes secundus*) and Sgr (*Schistocerca gregaria*).
