# Supplementary material for: Comparative Transcriptomics of Fat Bodies between Symbiotic and Quasi-Aposymbiotic Adult Females of Blattella germanica with Emphasis on the Metabolic Integration with Its Endosymbiont Blattabacterium and Its Immune System
Source: Int J Mol Sci. 2024 Apr 11;25(8):4228. doi: 10.3390/ijms25084228 (PMC11050582; doi:10.3390/ijms25084228)
Supplement: Supplementary file 1 [file ijms-25-04228-s001.zip › Table S2.pdf]

Comparative Transcriptomics of Fat Bodies Between Symbiotic and Quasi-Aposymbiotic Adult Females of *Blattella germanica* with Emphasis on the Metabolic Integration with its Endosymbiont *Blattabacterium* and Its Immune System

Francisco J. Silva, Rebeca Domínguez-Santos, Amparo Latorre and Carlos García-Ferris

Table S2. Endosymbiont load of pooled quasi-aposymbiont and control fat bodies determined by qPCR. *Blattabacterium* load was quantified by the copy number of gene *ureC* (only present in *Blattabacterium*) per ng of fat body DNA of the three fat bodies pooled in each sample. Abbreviation: q-Apo (quasi-aposymbiont).

Pooled Sample	Number of Copies of Gene *ureC*/ng of Fat Body DNA
q-Apo (pool 1)	5.73
	48.53
	13.33
q-Apo (pool 2)	4.43
	Below the limit of detection
	Below the limit of detection
q-Apo (pool 3)	Below the limit of detection
	Below the limit of detection
	Below the limit of detection
q-Apo (pool 4)	Below the limit of detection
	Below the limit of detection
	Below the limit of detection
Control (pool 1)	1.03×10^5
	1.37×10^5
	1.07×10^5
Control (pool 2)	1.63×10^5
	1.28×10^5
	1.21×10^5
Control (pool 3)	1.21×10^5
	2.20×10^5
	2.58×10^5
Control (pool 4)	2.24×10^5
	1.15×10^5
	1.09×10^5
